# Gamification improves antidepressant effects of cognitive control training—A pilot trial

**DOI:** 10.3389/fdgth.2022.994484

**Published:** 2022-10-21

**Authors:** Simone Weller, Philipp A. Schroeder, Christian Plewnia

**Affiliations:** ^1^ Department of Psychiatry and Psychotherapy, Tuebingen Center for Mental Health, University Hospital of Tuebingen, Tuebingen, Germany; ^2^ Department of Psychology, University of Tuebingen, Tübingen, Germany

**Keywords:** cognitive control, gamification, depression, cognitive control training, digital intervention, APP

## Abstract

**Objective:**

Computerised cognitive trainings have been put forward to improve control over negatively biased information processing and associated depressive symptomatology. Yet, disease-related impairments of motivation and endurance, as well as insufficient accessibility hinder use of this promising therapeutic opportunity. Here, we developed an app (*de:)press^©^*) that utilizes a cognitive control training (paced auditory serial addition task) enriched with gamification and information elements. We compared a six-week training with *de:)press^©^* to a non-gamified version (active control group).

**Methods:**

Thirty-two depressed participants were included. Each received either *de:)press^©^* or the non-gamified version and was instructed to train three times per week for two weeks. Afterwards (four weeks) they were free to train at their own discretion. Depression severity was assessed during training and two follow-up sessions. Primary endpoint was defined as difference between groups [change of Montgomery-Åsberg Depression Rating Scale (MADRS)] four weeks after end of training.

**Results:**

Depression severity decreased in both groups. At primary endpoint, MADRS scores were significantly lower in the *de:)press^©^*-group compared to the control group. No differences were observed at three months' follow-up. Intervention usability was consistently rated positively. Participants who had trained with *de:)press^©^* maintained the recommended training frequency without further prompting. Besides transient fatigue or frustration, no adverse effects were observed.

**Conclusion:**

This pilot demonstrates that gamification and information elements can substantially increase cognitive control training efficacy in alleviating depressive symptoms. Moreover, it provides first evidence for the feasibility and efficacy of *de:)press^©^* as an add-on intervention to treat depression.

**Clinical trial registration:**

The study is registered under ClinicalTrials.gov, identifier: NCT04400162.

## Introduction

Major depressive disorder (MDD) is a very common cause of morbidity and mortality that presents with low mood, loss of joy, hopelessness, lack of motivation, brooding, and other symptoms (1). Standard and mostly effective treatment approaches for MDD encompass psychotherapy, medication, and brain stimulation. Nevertheless, insufficient symptom relief remains a significant therapeutic challenge. This clinically relevant proportion of therapy-resistant symptomatology suggests that the available standard treatment does not sufficiently consider the pathophysiological variability (2), is not yet targeted enough or is underutilised due to lack of tolerance, high treatment costs, limited mobility, long waiting lists, lack of motivation, and concerns regarding stigma and privacy (3, 4). An expansion of therapeutic options would therefore be highly desirable.

Recent comprehensive evidence demonstrates that depression is linked with a wide range of cognitive deficits which for instance are indicated by dysfunctions in executive control, working memory, and processing speed (5). These impairments substantially affect quality of life (6) and represent a critical mediator of the association between depression and impaired psychosocial functioning (7). Even more importantly, this attenuated cognitive control (CC) is a critical factor in the development and maintenance of depression by means of a more salient experience and also preferential processing of negative information (*negativity bias*) (8–11). Consistent with Beck's cognitive model of depression (12, 13) attentional resources are withdrawn from external environment and predominantly allocated to negative internal experiences resulting in symptoms of depression (e.g., sadness, rumination, loss of motivation, hopelessness) (14). Moreover, the negative interpretation and negatively biased attention constitutes a feedback loop with mutually reinforcing subjective and behavioural symptoms. Therefore it can be derived that negatively biased cognition is not only a symptom of the acute depressive state but also a key pathophysiological factor. Consistently, studies with pharmacological (15), psychological (16), and neuromodulatory (17) interventions indicate that most of the effective treatments are linked with the normalisation of these biases (18) and an improvement of emotion regulation capacities (19), suggesting that improving CC—and in turn balancing out negativity biases—can be a viable addition to treatment. From the neurophysiological perspective, impaired cognitive control in depression is linked with a decreased prefrontal top-down regulatory influence on bottom-up activity (e.g., amygdala, hippocampus, and cingulate cortex) (8).

Based on this notion, cognitive control trainings (CCT) have been put forward as new ways to improve CC on negatively biased information processing and the associated depressive symptomatology (10, 20–22). Additionally, correcting the processing of such information may prove an effective tool for secondary prevention (23–26). In sum, CCT can be considered as a promising new tool for a multi-dimensional individualised treatment of MDD.

Training of cognitive or behavioural skills harnesses neuroplasticity to achieve clinical gains. It is assumed, that *via* constant and targeted exercise, critically weakened brain circuits will be strengthened and the associated control mechanisms will be restored. Therefore, to support clinically relevant and meaningful adaptive neuroplasticity, systems neuroscience-based circuit-specific trainings should be especially promising (27).

Yet, the number of clinical trials is small and hampers the drawing of conclusions on clinical utility of CCT (26). Clinical evidence for the efficacy of CCT predominantly comes from smaller laboratory studies, often with analogue mild depressed samples showing mixed results—full-scale controlled clinical trials with MDD patients are scarce. Nevertheless, recent meta-analyses indicate a small to medium effect size of CCT on mood and cognitive symptoms in MDD (28, 29). Naturally, methodological concerns must also be considered: nonspecific factors including patients' expectancy, engagement, novelty, and motivation (20) regarding the presented intervention may support efficacy of CCT. While these elusive factors cast doubt regarding the concrete mechanism of action, it has to be considered that, among others, environmental enrichment (30), reward (31), novelty (32) and background network activity (33) represent critical elements of the complex conditional structure within which adaptive neuroplasticity exists. Depending on the research question, these factors should be thoroughly assessed in future studies. For example, lack of motivation and decreased frustration tolerance can inhibit successful implementation and thus lower the effectiveness of CCT for the treatment of depression. Supporting and strengthening user engagement as well as training adherence may substantially improve efficacy (34, 35).

To address these challenges, we utilised gamification principles such as integrating psychoeducative elements, unlockable levels and progression tracking to enrich a digital and individually adaptive training paradigm: the *Paced Auditory Serial Addition Task* (PASAT) (36). This task has shown to have beneficial potential in supporting the treatment of depressive symptomology (37–40). Originally used for neurological testing (36), it was later applied in depressed patients as they exhibit decreased function of CC networks and re-activation of these networks can enhance cognitive functioning (40). The PASAT requires continuous attention, challenges the brain's processing speed by presenting stimuli with the individually determined minimal inter-stimulus interval, and trains the participant's ability to overcome distractions from negative feedback. The PASAT has shown to be quite demanding, monotonous, and sometimes frustrating (41, 42). It transiently induces negative affect (43, 44) as well as mental stress, indicated by increased cortisol levels (45). For best effects, this CCT needs to be performed on a regular basis over the course of at least several weeks. It can be assumed that the integration of gamification elements into the PASAT training (35) will likely improve the clinical feasibility and efficacy of CCT in the treatment of MDD. Various forms of gamification, i.e., the use of gaming elements in non-game contexts (46), were introduced to the PASAT to aid motivation and adherence to the task (47), resulting an easy to use mobile app (*de:)press^©^*). Each element we used (for a list see Table 1) can be categorised into one of five main dimensions of gamification: purpose, feedback, ownership, challenge, reward (46, 48–50). Providing the user with an elaborative context on the working mechanisms of a task has shown to not only add purpose to the training, but also allowing participants to set their own goals on what they want to achieve, which finally in itself is meant to increase motivation (51). The addition of feedback on performance creates a form of reinforcement, further fostering adherence to stick to the training paradigm (47).

**Table 1:** Comparison between the apps that CG and IG received.

Topic	Control group (CG)	Intervention group (IG)
Setting	Apart from PASAT instructions, there was no further explanation on reasoning or mechanisms of the task within the app. However, similarly to the IG group, the CG group was briefly given information on why the PASAT was chosen for this study.	A narrative that encapsulated the PASAT and its working mechanisms in a meaningful setting. Participants were taught about the biological and psychological background of depression aetiology (and possible supportive treatment options). This included artwork and other design options of the app.
Meaning and purpose	No additional information was given.	This provided participants with a theme that elaborated on the training's purpose: both in helping to improve their quality of life as well as giving them the opportunity for expressing feedback on game development and steering it in a useful, user-oriented direction. Participants were encouraged to browse through the different areas within the app and thus explore more of the background information on their own.
Progression	No feedback on progression was given.	The group was able to see their training progression over time *via* animated graphs. This was done to create an incentive to keep up with the training schedule and foster interest in continuing. Participants were made aware that drops in performance should be expected and to not be discouraged by them.
Levelling	No levels to unlock (ascending keyboard layout only).	If keeping up with the training schedule, participants could unlock further difficulty levels (ascending, descending, randomised keyboard layout) while the task itself remained the same. We included them to prevent ceiling effects in task performance, reset muscle memory, and increase cognitive load. Participants were allowed to switch freely between the unlocked levels during each training session.
Immediate feedback	Red or green screen after each trial, indicating whether the last response was wrong or right.	Identical to CG.
Long-term feedback	None.	Animated graphs on performance and training count, unlocking of achievements, interactions with avatars (see following lines).
Achievements/rewards	None.	When keeping up with the training schedule, participants unlocked up to 10 achievements (e.g., psychoeducation and information on the brain, the task, MDD, etc.). These achievements were also used to strengthen the “setting” and “meaning and purpose” aspect of the app.
Avatar	None.	An animated avatar acted as a “training companion” by guiding participants through the app, appearing in crucial screens, and visualise key components in the respective screens.

To test feasibility and efficacy of *de:)press^©^* training in addition to standard treatment of MDD, we compared the gamified training to the same PASAT training paradigm without any gamification elements in a randomized controlled pilot trial. We expected sustainable reduction of MDD symptoms 4 weeks after a 6-week intervention phase.

## Materials and methods

### Ethics

The study was conducted in accordance with the declaration of Helsinki on the ethical principles of medical research involving human subjects. The ethics committee of the University Hospital Tübingen gave their positive vote on the protocol for this study.

### Participants and study groups

In a previous study that used the PASAT (40), large reductions in depressive symptoms and rumination (*d* = 1.26 / *d* = 1.28) were observed. To reproduce these effects with high power (*α* = 0.05; 1—*β* = 0.95), a total of *n* = 11 participants per group would be needed. This relatively small sample size would be sufficient to provide first effect size estimates of the gamified training and to allow for larger high-quality follow-up trials. To ensure more robust results, we increased that number and did enrol 16 participants per group.

Out of 55 persons 32 adult applicants (female and male) met the inclusion criteria. They were diagnosed with acute or chronic recurrent major depressive disorder (MDD) and were recruited through the University of Tübingen mailing lists, posters, and flyers displayed around campus. Participants were randomly assigned to either the *control group* (CG) or the *intervention group* (IG; *n* = 16 respectively). While the CG received the “bare” CCT without any gamification elements, the IG received the same CCT enriched with several motivational and educational elements (see section *Gamified Cognitive Control Training: de:)press^©^*).

*Inclusion criteria:* at least 18 years old, ability to give consent, appropriate knowledge of German (at least CEFR level B), current MDD (F32, F33) as diagnosed by the Mini-International Neuropsychiatric Interview, light to moderate manifestation of the MDD as defined by a MADRS score between 10 and 34 at the time of the first study visit, either no or stable antidepressant/psychoactive medication (since at least 6 weeks before inclusion in the study).

*Exclusion criteria:* psychotic symptoms or schizophrenia, dementia, a history of epilepsy, other mental disorders (current or in the past—an exception to this rule were anxiety phobic or panic disorders as they often occur concurrently with MDD), suicidality.

### Gamified cognitive control training: *de:)press^©^*

As a CCT we chose the PASAT. This task has proven to be a frustrating challenge regardless of cognitive state as it adapts to a participant's performance and provides a continuous cognitive challenge (36, 42). Participants were each given a tablet computer which had the PASAT installed in a “kiosk mode”, allowing only interaction with the task and no other tablet functions. Hence, for this study, participants were not required to use their own devices or go through the install process themselves. No updates were deployed over the course of the study, each participant received the same final version. The app is a native Android app and registered under the name *de:)press^©^*. It requires at least Android 8.1.0 and 2 GB of RAM and was developed with Android Studio. All data collected and processed by the app remained on the device until exported to perform statistics, no internet connection was needed.

**Figure 1:**
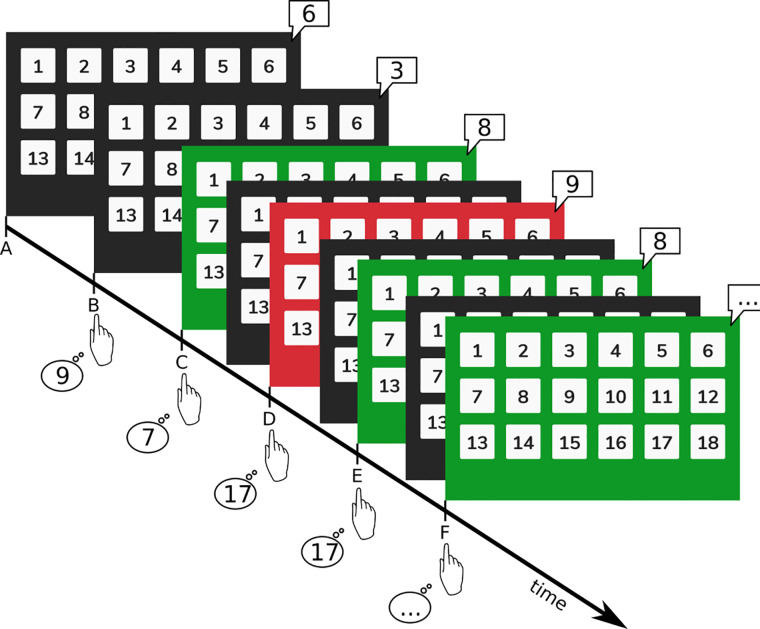
Visual representation of the PASAT. Participants heard single digit numbers (here shown in speech bubbles) from the tablet's speakers and were asked to add the last digit to the second-to-last digit (e.g., digits at timepoints A + B, B + C, C + D, and so forth). Numbers were presented with an initial interval of 3 s. Answers were then given on the keyboard. For correct answers the screen briefly flashed green, for wrong answers the screen flashed red and then immediately return to a dark background. This feedback was given *concurrently* to the following digit presentation (e.g., green feedback at E refers to the correct result given for the addition of C + D).

**Figure 2:**
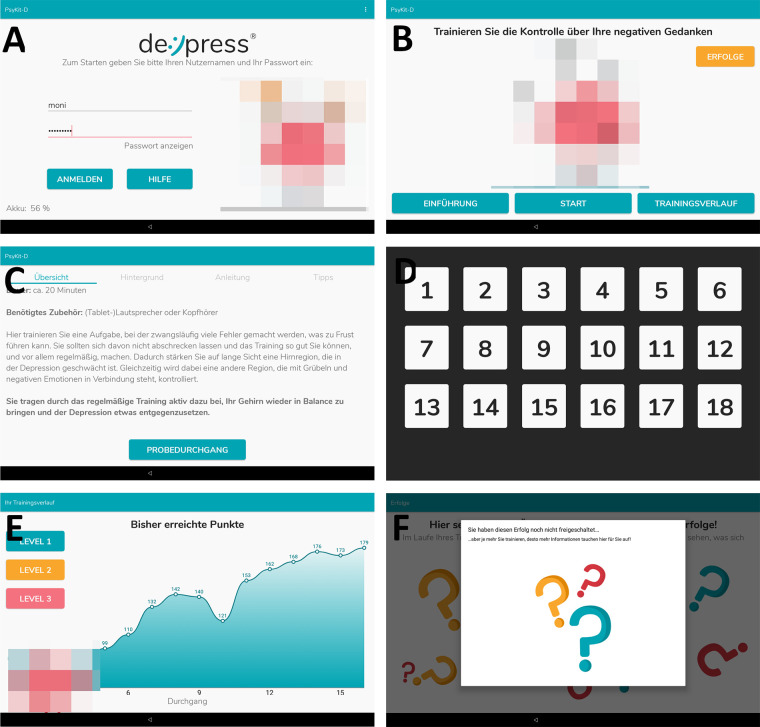
Screenshots from the IG app. (**A**) Login screen. (**B**) Training hub, from which participants were able to chose which area of the app they wanted to explore. (**C**) Introduction and instructions for the training. Furthermore, psychoeducative information was provided. (**D**) Keyboard layout during training. This layout shows the first level with an ascending button layout. (**E**) Progression graph that shows the number of correct responses within each training session (“highscore”). (**F**) Achievements that can be unlocked during training. In this screenshot no achievements have been unlocked yet. The CG app consisted of screens A (without avatars), (**C,D**).

Participants were presented with a continuous auditory stream of single digit numbers with an initial *inter-stimulus interval* (ISI) between digit presentations of 3 s. Participants were then instructed to add the last two digits they heard to each other: the current digit and the digit that was presented directly before it. Answers were given by pressing the respective answer button shown on the screen. See Figure 1 for a visual representation of the task and Figure 2 for screenshots of the app. In general, to successfully perform in this CCT, participants must stay focused and not let themselves be distracted by errors, the provided feedback of their recent addition (green screen for correct responses, red screen for wrong, late, or non-responses), or negative thoughts. Four consecutively correct answers shortened the ISI by 0.1 s, four consecutively wrong answers lengthened the interval by the same amount. Consequently, the PASAT adapted to individual performance and provided a continuous challenge. The task was divided into three blocks, each block lasting five minutes. Blocks were intercepted by short breaks (30 s) and an initial countdown of 30 s, amounting to a training duration of 16 min 30 s per session. The ISI was carried over from block to block, however it was reset for each new training session.

### Gamification elements of the cognitive training

To specifically test the additional effects of gamification elements and mediation of purpose-driven motivation, the app was kept as minimal as possible for the CG, while the IG received the enriched training. Table 1 highlights the main differences between the two versions. Please refer to Figure 2 for screenshots of the app.

### Study timeline

**Figure 3:**
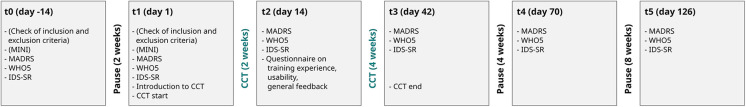
Study timeline showing the content of each session.

Before taking part in the study, all participants gave written informed consent. They were to attend 5 sessions (t1–t5, see Figure 3), each during which they answered questionnaires and took part in psychological interviews. Half the participants of each group attended an additional session 2 weeks before start of the training (t0). This was done to evaluate possible changes in depressive symptomatology prior to our intervention. In t1, all participants were given the tablet with the CCT installed on it. In the 2 weeks between t1–t2 they were instructed to train at least every second day (equal to 3 times per week). From t2–t3 (4 weeks) they were asked to train as often as they saw fit. In t3 they returned the tablets. Four weeks after end of training (t4) main outcomes were assessed, final follow-up was 12 weeks after end of training (t5).

### Questionnaires

#### Montgomery-Åsberg depression rating scale (MADRS)

The MADRS (52) is a semi-structured interview to assess MDD severity. The assessment period is the previous week and consists of 10 items, each of which is rated on a 7-point scale from 0 to 6 by a trained psychologist. The psychologists who performed the ratings were blind to the intervention that each participant received. The MADRS is considered the gold standard for measuring the severity of depressive symptoms (53), especially because its high sensitivity to changes.

#### Inventory of depressive symptomatology, self-report version (IDS-SR)

The Inventory of Depressive Symptoms (IDS-SR) is a 28-item self-report depression scale utilised to determine the severity of depressive symptoms (54, 55).

#### WHO-Five-Well-Being Index (WHO5)

The WHO5 is a short (5 items) self-report questionnaire designed to assess overall well-being. It has been recommended by the World Health Organization as a screening questionnaire for depression and is suitable as an outcome for clinical trials (56).

#### Usability and general user feedback

Participants answered 18 custom questions regarding the usability, stability, and design of the software, as well as the training paradigm itself. Additionally, there were 7 free-text questions for participants to give feedback and recommendations on the training and software.

### Statistical analyses

All statistical analyses were done with IBM SPSS version 27 (57) and R version 4.0.4 (58). The factors used in the statistical models are defined as such: *group* (CG or IG) and *time* (session during which the measurement was taken, t0–t5).

We used t-tests to analyse differences between study samples (measured at t1) and usage frequency (measured at t3). Distributions within groups (sex, current pharmacotherapy, current psychotherapy) were measured *via* fisher's exact tests. Possible changes during the pre-training phase (t0–t1) were measured *via* t-tests. A linear mixed model (LMM with restricted maximum likelihood estimation) was used to analyse the development of depression symptoms from start of training to primary endpoint (t1–t4) as this method is most robust against single missing sessions (see following sections for drop-out rates). Fixed effects were scores of the respective questionnaire, group, session during which the measurement was taken, the interactions between scores and session, and baseline scores of the questionnaire. Random effects were measurement timepoint and individual subject. Post-hoc analyses of t4 (primary end point) and t5 (follow-up) were done *via* t-tests.

## Results

**Figure 4:**
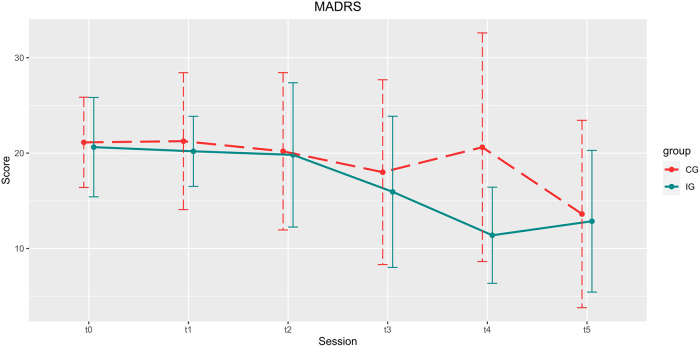
Development of the MARDS scores over the course of the study. Depicted are means and standard deviations (SD). Participants of the IG showed significantly higher improvement in the reduction of depression scores up until 4 weeks after the end of the training (t4).

**Figure 5:**
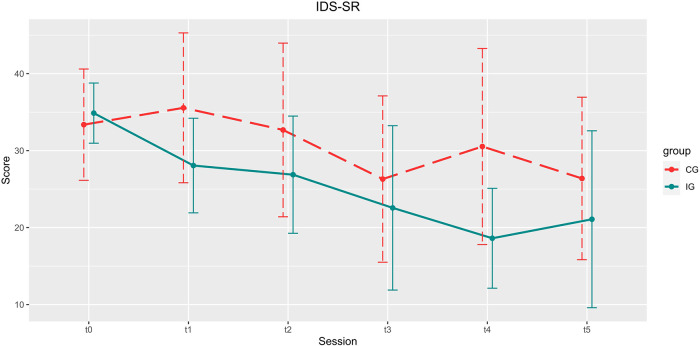
Development of the IDS-SR scores over the course of the study. Depicted are means and standard deviations (SD).

**Figure 6:**
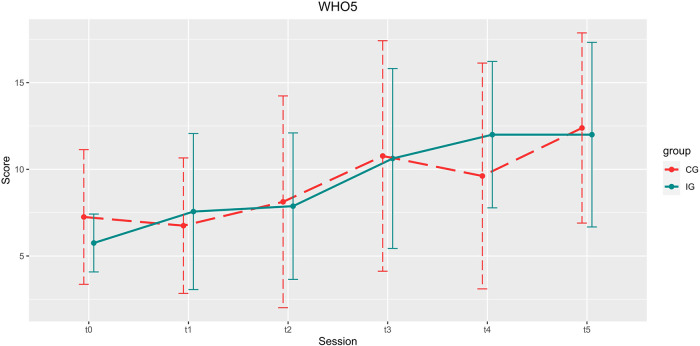
Development of the WHO5 scores over the course of the study. Depicted are means and standard deviations (SD).

See Table 2 for an overview on demographic data and Table 3 for the scores of each interview and questionnaire. See Figures 4–6 for a visual representation of primary and secondary outcomes.

**Table 2:** Demographic and general data.

Measure	Control Group (CG)	Intervention Group (IG)
Age range (min-max)	18–63	21–76
Mean age (±SD)	30.00 ± 13.33	40.19 ± 16.63
Sex (f/m)	9/7	10/6
Psychotherapy (y/n)	9/7	6/10
Medication (y/n)	9/7	5/11
Mean number of trainings (t1–t2)	5.69 ± 4.11	6.47 ± 1.92
Mean number of trainings (t2–t3)	8.81 ± 8.39	12.80 ± 10.61
Mean number of trainings (total)	14.50 ± 11.39	19.27 ± 10.92

**Table 3:** Scores for questionnaires and interviews per session. Shown are mean and standard deviations (±SD).

Timepoint	CG			IG		
	MADRS	IDS-SR	WHO5	MADRS	IDS-SR	WHO5
t0	21.13 ± 4.73	33.36 ± 7.23	7.25 ± 3.88	20.62 ± 5.21	35.86 ± 3.91	5.75 ± 1.70
t1	21.25 ± 7.19	35.56 ± 9.74	6.75 ± 3.91	20.19 ± 3.67	28.06 ± 6.15	7.56 ± 4.50
t2	20.19 ± 8.25	32.69 ± 11.28	8.13 ± 6.12	19.81 ± 7.56	26.88 ± 7.61	7.88 ± 4.22
t3	18.00 ± 9.69	26.31 ± 10.80	10.77 ± 6.65	15.94 ± 7.93	22.56 ± 10.68	10.63 ± 5.19
t4	20.62 ± 11.99	30.54 ± 12.74	9.62 ± 6.51	11.38 ± 5.04	18.62 ± 6.49	12.00 ± 4.22
t5	13.62 ± 9.83	26.38 ± 10.56	12.38 ± 5.49	12.86 ± 7.43	21.08 ± 11.49	12.00 ± 5.33

### Overview of the study sample and pre-training phase (t0–t1)

#### Study sample (t1)

There were no differences between groups in age (*t*(30) = −1.912, *p* = 0.065, *d* = −0.676). The distribution of sexes (*p* = 1), participants taking any form of psychiatric medication (*p* = 0.285), and participants undergoing psychotherapy (*p* = 0.479) was equal in both groups.

#### Pre-training phase (t0–t1) and baseline (t1)

In the waiting groups there were no significant changes from t0 to t1 in either MADRS or WHO5. However, IDS-SR scores in the IG lowered significantly during this period. See Table 4 for an overview of the statistics.

**Table 4:** Statistical analysis of the pre-training phase (t0t1).

	CG	IG
MADRS	t (7) = 0.751, *p* = 0.477, *d* = 0.265	t (7) = 0.632, *p* = 0.547, *d* = 0.224
IDS-SR	t (7) = 0.081, *p* = 0.938, *d* = 0.028	t (7) = 2.799, *p* = 0.027, *d* = 0.990
WHO5	t (7) = 0.000, *p* = 1.000, *d* = 0.000	t (7) = −1.910, *p* = 0.098, *d* = −0.675

There were no significant changes in either group during this phase except for IDS-SR scores in the IG, which lowered significantly.

Scores at start of training (t1) were not significantly different between CG and IG for MADRS [*t*(30) = 0.527, *p* = 0.603, *d* = 0.186] and WHO5 [*t*(30) = −0.545, *p* = 0.590, *d* = −0.193]. Yet, the IDS-SR was significantly higher for IG [*t*(30) = 2.605, *p* = 0.014, *d* = 0.921], denoting a higher perceived depressive symptomatology within this group.

### Training and primary endpoint (t1–t4)

#### Primary endpoint: MADRS

Compared to the CG, gamified training led to a significantly stronger alleviation of depressive symptoms during the intervention and the following 4 weeks [*group* x *time*: *t*(85) = −2.395, *p* = 0.019, B = −2.652, *β* = 1.107]. The main effects *group* [*t*(85) = 1.881, *p* = 0.063, B = 4.258, *β* = 2.264] and *time* [*t*(85) = −0.101, *p* = 0.920, B = −0.080, *β* = 0.794] were not significant. Post-hoc comparison between IG and CG at t4 (CG: 20.62 ± 11.990, IG: 11.38 ± 5.042) showed a significant superiority of the gamified training [*t*(16.116) = 2.559, *p* = 0.021] with large effect size (*d* = 1.004). The comparison between MADRS scores in t1 and t4 for the IG shows a significant improvement [*t*(12) = 5.503, *p* < 0.001, *d* = 1.526], which is not found in the comparison between t1 and t4 in the CG.

#### IDS-SR

There was a significant reduction of the total score for all patients [main effect *time*, *t*(84) = −1.984, *p* = 0.051, B = −1.870, *β* = 0.942], but no significant interaction between *group* and *time* [*t*(84) = −0.843, *p* = 0.401, B = −1.088, *β* = 1.290], and no main effect of *group* [*t*(84) = 0.962, *p* = 0.339, B = 2.311, *β* = 2.403].

#### WHO5

There was no significant increase in WHO5 scores in either group for main effect *time*, *t*(84) = 1.480, *p* = 0.143, B = 0.815, *β* = 0.551), between *group* x *time* [*t*(84) = 1.022, *p *= 0.310, B = 0.756, *β* = 0.739] or main effect *group* [*t*(84) = −1.143, *p* = 0.256, B = −1.434, *β* = 1.255].

### Follow-up (t5)

#### MADRS

Twelve weeks after the end of the intervention, the total sample showed a significant reduction in MADRS score compared to baseline [*t*1: 20.67 ± 5.428, t5: 13.22 ± 8.505; *t*(26) = 4.281, *p* < 0.001, *d* = 0.824].

#### IDS-SR

There was a significant decrease in self-reported depressive symptomatology for the whole group [t1: 32.56 ± 9.106, t5: 23.84 ± 11.116; *t*(24) = 4.256, *p* < 0.001, *d* = 0.851].

#### WHO5

There was a significant increase in overall well-being for the total sample [t1:7.04 ± 4.449, t5: 12.20 ± 5.299; *t*(24) = −4.151, *p* < 0.001, *d* = −0.830].

### Usage

#### Usage frequency

From t1–t2, during which the participants were instructed to train at least 3 times per week, IG trained 6.46 ± 1.92 times on average, CG 5.69 ± 4.11 times on average. During the following four weeks (t2–t3), participants were asked to exercise as often as they found helpful. In the IG, this was on average 12.80 ± 10.61 times, in the CG amount of training sessions was 8.81 ± 8.39 times. Hence, we can conclude that the IG maintained the recommended training frequency of 3 times per week without further prompting. However, the difference in the number of total training session between *de:)press^©^* and the non-gamified PASAT was not statistically significant in this sample (19.267 ± 10.924 vs. 14.500 ± 11.390; *t* = −1.188; *p* = 0.245, *d* = −0.427).

#### Usability

Regardless of intervention type, the training was perceived positively: reliability and overall feedback of the software reached 82.81% (where +100% corresponds to a maximally positive evaluation, 0% to a neutral evaluation, and −100% to a maximally negative evaluation). Design and usability reached 74.06%. The training itself scored 48.05%. In a questionnaire on the intuitive use of the system no differences were found between the groups.

#### Side effects

No severe side effects were reported. Fatigue was occasionally reported as occurring directly after the training, which is an expected outcome of a demanding cognitive training. Participants also reported frustration during the task, however this subsided the longer the training was continued.

#### Drop-out rates and aborted training sessions

Some subjects were not able to attend certain sessions but might have been available at later sessions again. During the training phase no subject dropped out in the IG but 2 in the CG (missing values at t3). At the 4 weeks follow-up assessment (t4) 3 subjects were not available in either group. At the 12-weeks follow-up (t5) data from 2 subjects in the IG and 3 in the CG were missing. No training sessions were terminated prematurely.

## Discussion

With this study, we tested the feasibility of an app-based, gamified PASAT training (*de:)press^©^*) and its effect on depression severity. To focus on the relevance of gamification and to allow for a meaningful effect-size estimation of *de:)press^©^*, a non-gamified PASAT was used as active control condition. With *de:)press^©^*, we found a greater decrease in depressive severity (MADRS) during and up to four weeks after the intervention. Additionally, both depression severity and usage frequency were more stable in the enriched compared to the control version of the CCT. These findings indicate that *de:)press^©^* has the potential for an adjunctive treatment of depression and that the antidepressive effect of this gamified digital health intervention may even surpass the PASAT training without the added motivating, playful, and informative elements. However, at follow-up, 12 weeks after the end of the training phase, a substantial reduction of depressive symptoms was visible in both groups. The usability of the intervention was consistently rated positively by its users. Except for slight occasional fatigue and transient frustration, no adverse events or side effects were observed.

### Improvement of depression with PASAT-training

The superiority of *de:)press^©^* compared to the active control condition (reduction by 9.2 MADRS points, 45%) as well as to baseline (reduction by 8.8 MADRS points, 44%) in a real-world sample of patients with depression is clinically meaningful (59) and is maintained for up to 3 months after intervention. This beneficial effect is in line with previous findings in studies that applied the PASAT to alleviate symptoms of depression (37–40). However, with this it is not shown that other that other forms of CCT cannot also be effective. Nevertheless, by simultaneously challenging cognitive core features of depression (60) such as: deficits in working-memory (61), attention (62), processing speed (63), cognitive effort (64), and the control of negative feedback (25) at the individual performance maximum, *de:)press^©^* allows for a retraining of brain networks that are critical for the development and maintenance of MDD. Given the goal of maximizing the clinical effectiveness, simultaneous activation of the various processes seems most promising. However, the specific contribution of each of these processes to antidepressant efficacy, remains to be elucidated.

### Facilitative effects of gamification on training

Our data show that depression adapted gamification as well as the comprehensive and patient-oriented information about the purpose of the training can substantially enhance anti-depressive features of the CCT. So far, gamification was not systematically used to enhance the efficacy of PASAT training in the treatment of MDD, and evidence regarding the facilitatory effect of gamification in mental health apps is mixed (65–67). Improving motivation and frequency of use through engaging and motivational elements could support those patients who have deficits specifically in this area. Notably, depression adapted gamification goes beyond the mere inclusion of game elements but encompasses meaning, psychoeducation, and broader support (see Table 1) derived from clinical experience and patient feedback. While the training proved to be a challenge for participants, the vast majority kept up with the training schedule, and the few dropouts were caused by external factors such as sudden family issues or non-related illnesses. It can therefore be assumed that gamification makes cognitive training programs more acceptable and increases the motivation to get it done. However, neuroplasticity-enhancing factors of gamification should also be considered. Beneficial effects of reward (31), motivation and attention (27, 68), and environmental enrichment (69) may additionally support adaptive reorganization and recovery. In *de:)press^©^*, a pragmatic, user- and usability-oriented mixture of these factors is used, as the app tries to utilise these gamified elements without overwhelming the user with too many options. It can be assumed that these factors also, by facilitating adaptive reorganization, contributed to the antidepressant effect and its sustainability. Accordingly, with added gamification elements more patients may benefit from the intervention, while also benefitting more from training. Unfortunately, this question cannot be answered based on the present sample.

### Need for long-term training and follow-up

Of note, the specific efficacy of *de:)press^©^* in the reduction of depressive symptoms is particularly visible 4 weeks after the end of its use. Considering the assumed mechanisms of action, this is not surprising. On the one hand, similar to physical exercise, it takes a while before cognitive training produces benefits that are recognizable for the trainee; on the other hand the PASAT-training as used in *de:)press^©^* aims to improve control of negative and stressful information (40, 43)—a process that may take time to induce a clinically tangible impact (70). The need for a sufficiently long training and observation period is illustrated by a recently published study indicating a lack of antidepressant effect of PASAT training compared to a sham-training control condition. Here, a non-gamified PASAT intervention comprising 10 training session within 2 weeks in the context of an inpatient treatment did not yield superior effects on depression severity. However, an exploratory analysis revealed significantly higher levels of subjective well-being in the active compared to the sham group at 1-year follow-up (71). This is consistent with prior studies showing significant between-group differences in depression symptomatology only at 3 months follow up after PASAT training (70). It indicates that training effects on depressive symptoms do not become visible immediately after the end of training but after a longer period of time. Regarding the amount and the spacing of training, available studies on CCT point to an optimum of 10–15 h of training spanned over several weeks (20). However, in the case of depression, a limited endurance of the patients must be considered. Consistently, most interventions elicit positive effects if a long enough training period is chosen (35, 38, 72). In this context, our training schedule comprising a 6 week intervention with three trainings per week proved to be adequate.

### Limitations

Several limitations should be considered, most of which will be addressed in the follow-up study. First, an increased number of participants would have been beneficial for the stability of the findings. However, the Corona pandemic hindered recruitment.

While we saw beneficial effects of the training, due to the multimodality of the task itself and the surrounding gamification elements, it remains to be seen which factors contributed most (and in which way) to recovery. This could be targeted by strategically comparing versions of the app that differ in their number of gamified elements and how they are implemented.

Within this study, we compare two active groups against each other. While this allows us to draw conclusions on how either of the app versions worked, we have no comparison to treatment as usual. In an ongoing follow-up study, we will address these points.

## Conclusions

This pilot study shows the feasibility and usability of *de:)press^©^* as an adjunctive treatment option of MDD by demonstrating that participants adhere to the training paradigm and show a lasting decrease in depressive symptoms. Based on the notion that good mental health is an active process (18), *de:)press^©^* empowers, enables and encourages patients to regain cognitive control and thus effectively participate in a key aspect of overcoming their depression. By inclusion of depression adapted gamification elements and mediation of purpose-driven motivation the beneficial effects of CCT can substantially be enhanced.

## Data Availability

The raw data supporting the conclusions of this article will be made available by the authors, without undue reservation.
